# Feminization of the Blood–Brain Barrier Changes the Brain Transcriptome of *Drosophila melanogaster* Males

**DOI:** 10.3390/cimb47080626

**Published:** 2025-08-06

**Authors:** Danyel S. Davis, Warda Hashem, Chamala Lama, Joseph L. Reeve, Brigitte Dauwalder

**Affiliations:** Department of Biology and Biochemistry, University of Houston, Houston, TX 77004, USA; dsdavis6@cougarnet.uh.edu (D.S.D.); whashem@cougarnet.uh.edu (W.H.); chamalalama@gmail.com (C.L.); jlreeve@utexas.edu (J.L.R.)

**Keywords:** *Drosophila*, Blood–Brain Barrier, transcriptome, sex-specific

## Abstract

Beyond its crucial role as a tight barrier to protect the nervous system, the Blood–Brain Barrier (BBB) is increasingly being recognized for its physiological processes that affect brain function and behavior. In *Drosophila melanogaster*, the BBB expresses sex-specific transcripts, and a change in the sexual identity of adult BBB cells results in a significant reduction in male courtship behavior. The molecular nature of this BBB/brain interaction and the molecules that mediate it are unknown. Here we feminize BBB cells by targeted expression of the *Drosophila* female-specific master regulator TraF in otherwise normal males. We examined the effect on RNA expression in dissected brains by RNA sequencing. We find that 283 transcripts change in comparison to normal control males. Transcripts representing cell signaling processes and synaptic communication are enriched, as are hormonal mediators. These transcripts provide a valuable resource for addressing questions about BBB and brain interaction.

## 1. Introduction

*Drosophila* male courtship is well described. However, many questions remain about its regulation on a molecular level. Male courtship behavior in *Drosophila melanogaster* is regulated cell-autonomously by the overall sex determination hierarchy [1]. It is characterized by a series of sex-specific alternative splicing steps. As part of this cascade, a sex-specific master regulator, Transformer (TraF), controls the generation of DSX and FRU proteins by regulating the alternative splicing of their respective pre-mRNAs. In females, where functional TraF is present, DSXF is generated but no FRU protein is made. In males, the absence of TraF leads to the default splicing of *dsx*- and *fru* pre-mRNAs and the male specific DSXM and FRUM proteins are produced (for reviews see [2,3]). It is well known that the courtship behavior of *Drosophila* males is regulated by this cascade and ultimately *dsx* and *fru*, with dedicated neuronal circuits that express DSXM and/or FRUM proteins [4,5,6,7,8,9,10,11,12]. It has also become clear that non-neuronal cells, as well as tissues outside the brain play a significant role in the regulation of courtship. The fat body is a prominent example. The fat body is best known for its metabolic and secretory functions. In addition, it has been found that it produces numerous sex-specific proteins [13,14]. When fat body cells were genetically feminized by fat-body specific expression of the female TraF protein in otherwise normal males their courtship was significantly reduced, demonstrating that male-specific fat body factors regulate courtship [15]. It was shown for one of these sex-specific fat body proteins, Takeout, that it is present in the hemolymph, which is the circulating body fluid of insects. *takeout* mutants have reduced courtship [13] that can be rescued when wildtype *takeout* is expressed in different secretory cells, demonstrating that it acts as a secreted protein [15]. It is likely that other sex-specific fat body proteins act in a similar way. However, in order to interact with the brain and control behavior, circulating proteins need to negotiate the Blood–Brain Barrier (BBB). How circulating molecules interact with these cells and how this interaction regulates brain function is largely unknown. Because flies have an open circulatory system, the BBB surrounds the brain like a tight cap. Despite differences, BBB properties and many proteins are conserved between the vertebrate and the fly BBB [16,17,18,19,20,21,22,23,24]. 

The BBB is a selective barrier that regulates access to the brain. It is increasingly being recognized that it also plays a dynamic role in the regulation of brain function by peripheral factors. A prominent example is in development, where available nutrients in the hemolymph lead to changes in the BBB that result in a coordination of BBB growth and neuroblast proliferation, enabling the balanced growth of the larval brain [25,26,27]. 

Several adult behaviors have been shown to be regulated by physiological processes in the BBB. A pioneering study by Bainton et al. demonstrated that the response to ethanol and cocaine is dependent on signaling by the *moody* GPCR in the BBB [16]. Recent studies have found links between regulated BBB permeability and lipid trafficking in the regulation of sleep and suggested that these processes may be part of a sleep-need sensing system [28,29,30]. 

Intriguingly, the BBB has also been shown to regulate male courtship behavior. When the sex of BBB cells of otherwise normal males was genetically changed to female in mature flies their courtship behavior was significantly reduced, suggesting a regulatory role for male-specific BBB transcripts in the regulation of courtship [31]. Microarray profiling of BBB cells from males and females revealed numerous sex-specific transcripts in males and females [32]. What are the molecular changes following feminization that interfere with the normal function of the courtship circuits? To begin to address this question, here we characterize the brain transcripts that change upon BBB feminization by targeted expression of TraF in these cells. Expression of TraF has been shown to specifically feminize the cells in which it is expressed owing to the cell-autonomous nature of *Drosophila* sex determination [33,34], and we have previously shown that this BBB manipulation reduces male courtship [31]. Following targeted BBB feminization we performed RNAseq analysis of dissected brains (this includes the BBB) and compared males with feminized BBB to control males. We find changes in 283 transcripts that are candidates for mediating sex-specific functions of the BBB, providing a valuable resource for addressing questions about BBB and brain interaction. 

## 2. Materials and Methods

### 2.1. Fly Stocks

*SPG-GAL4/TM3,Sb (moody-Gal4)* [16,32] was a gift from Roland Bainton, UCSF. *w^1118^*; *P(UAS-RedStinger)4/CyO (BL 8546)* and *w*; *UAS-traF* (BL 4590) were obtained from the Bloomington *Drosophila* stock center, Indiana University, Bloomington, IN, USA “https://bdsc.indiana.edu/ (accessed on 12 July 2025)”. The *SPG-GAL4* and *UAS-traF* stocks were outcrossed with a *Cantonized w^1118^* stock for 10 generations prior to the experiment. *SPG-GAL4* and *UAS-traF* flies were crossed to yield *w^1118^*; *+*; *SPG-GAL4/UAS-traF* experimental flies. Controls were *w^1118^*; *+*; *SPG-GAL4/+* and *w^1118^*; *+ UAS-traF/+*.

### 2.2. Whole Brain Dissection and RNA Sequencing

Flies were grown in a 25 °C incubator in a 12 h light/12 h dark cycle on standard cornmeal/yeast food. Eclosing males were collected and kept in groups of 10-15 flies under the same conditions for 4 days and then dissected between ZT 5 and ZT 7 to control for significant circadian influence on gene expression. Equal numbers of flies originating from the same culture were dissected in each sitting for all genotypes, with all crosses reared under identical conditions. 4–5 days old male flies were shortly anesthetized on ice before dissection. Dissection was performed in ice cold 1 X Phosphate-Buffered Saline (PBS) on ice. The fly brains were dissected by using straight Dumont # 5 fine forceps (Fine Science Tools, Inc, Foster City, CA 94404-4824, USA). Once dissected, the brains were cleaned of cuticle debris, fat body and trachea. The cleaned brains were immediately transferred to a small droplet of Trizol reagent (~ 20 µL); (Invitrogen, Thermo Fisher Scientific, Waltham, MA 02451, USA), frozen on a sterile weigh boat on dry ice. The brains were then stored at −80 °C in a pre-cooled 1.5 mL Eppendorf tube until processed for total RNA extraction. At least 450 brains were dissected for each genotype and biological replica. RNA was extracted using the RNeasy mini kit (Qiagen, Germantown, MD 20874, USA) following the supplier’s protocol. Sequencing was performed at the *Baylor College Genomic and RNA Profiling Core* (Baylor College of Medicine, Houston, TX 77030, USA). Sample Quality was checked using a NanoDrop spectrophotometer and Agilent Bioanalyzer 2100. The Illumina TruSeq RNA v1 library preparation protocol was used to prepare samples for sequencing. A double-stranded DNA library was created using 2 ug of total RNA, preparing the fragments for hybridization onto a flowcell. cDNA was created using the fragmented 3’ poly (A) selected portion of total RNA and random primers. Libraries were created from the cDNA by first blunt ending the fragments, attaching an adenosine to the 3’ end and finally ligating unique adapters to the ends (For more information on this process, see below). The ligated products were then amplified using 15 cycles of PCR. The resulting libraries were quantitated using the NanoDrop spectrophotometer and fragment size was assessed with the Agilent Bioanalyzer. A qPCR quantitation was performed on the libraries to determine the concentration of adapter ligated fragments using a Bio-Rad iCycler iQ Real-Time PCR Detection System and a KAPA Library Quant Kit. 6pM of library was loaded onto a flowcell and amplified by bridge amplification using the Illumina Cluster Station instrument. A paired-end 75 cycle run was used to sequence the flowcell on an Illumina Genome Analyzer (GAII) Sequencing System. Samples were assigned the following numbers in sequencing: *w^1118^*; *SPG-GAL4/UAS-traF*: # 27978 and 27981; *w^1118^*; *SPG-GAL4/+*: 27977 and 27980; *w^1118^*; *UAS-traF/+*: 27979 and 27982.

### 2.3. Sequence Analysis 

We conducted a comprehensive RNA-Seq analysis to identify differentially expressed RNAs in *Drosophila melanogaster* samples. Our workflow encompassed data preprocessing, differential expression analysis, and R studio visualization. Raw paired-end FASTQ files were obtained from Baylor College of Medicine. Quality assessment confirmed high data integrity, with mapping rates exceeding 76% for genomic DNA and 80% for mRNA. Transcript abundance was quantified using Kallisto. The Kallisto index was generated from the reference transcriptome, and abundance estimates for each sample were produced. 

Differential expression analysis was performed in R studio (version 2025.05.1 (Posit Software, PBC, Boston, MA, USA)) using the DESeq2 package. Abundance estimates from the abundance.tsv files were extracted and compiled into a unified count matrix with transcripts as rows and samples as columns. Rows with zero counts across all samples were removed. Sample conditions were labeled as either experimental or control based on their biological grouping. The DESeqDataSET was created using the count matrix and metadata defining experimental condition, and the DESeq function was applied to compute foldchanges and statistical signifigance for each gene. Genes with an adjusted *p*-value (padj) less than 0.1 were considered significant. 

Comparison between RNA datasets was performed using the ‘inner join’ function of the dplyr package “https://dplyr.tidyverse.org/reference/mutate-joins.html, accessed on 12 July 2025”. GO analysis was performed using “https://www.flymine.org, accessed on 12 July 2025”. Heat maps were generated using “http://www.bioinformatics.com.cn, accessed on 12 July 2025” [35]. 

## 3. Results

**Figure 1:**
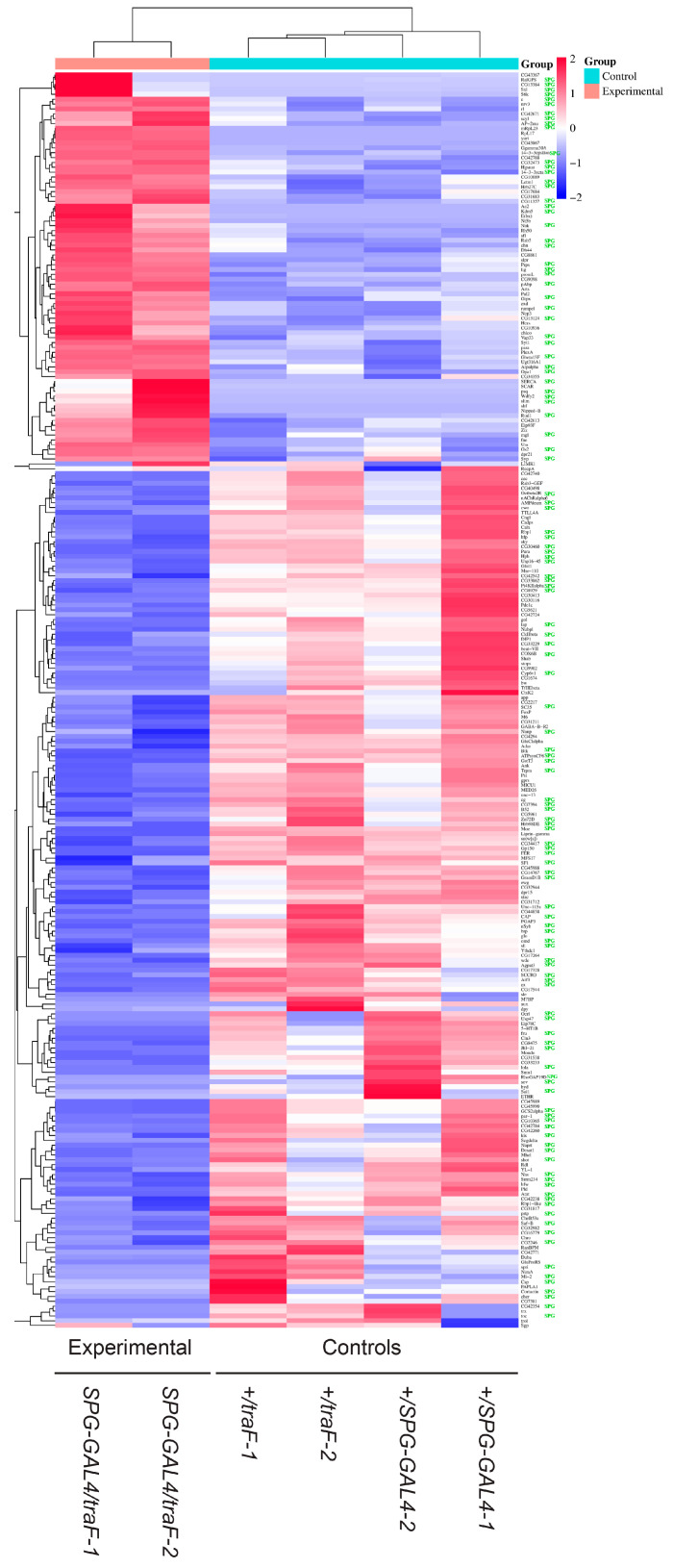
*Differentially expressed transcripts in response to BBB traF expression*. Heatmap showing transcripts with changes in gene expression in isolated brains of control males (*w^1118^*; *+*; SPG-GAL4/+ and *w^1118^*; *+ UAS-traF/+*, respectively) and males with feminized BBB (*w^1118^*; *+*; *SPG-GAL4/UAS-traF*). Red to blue shadings represent higher and lower relative expression levels, respectively. Transcripts identified as present in SPG cells (see below) are marked in green.

**Figure 2:**
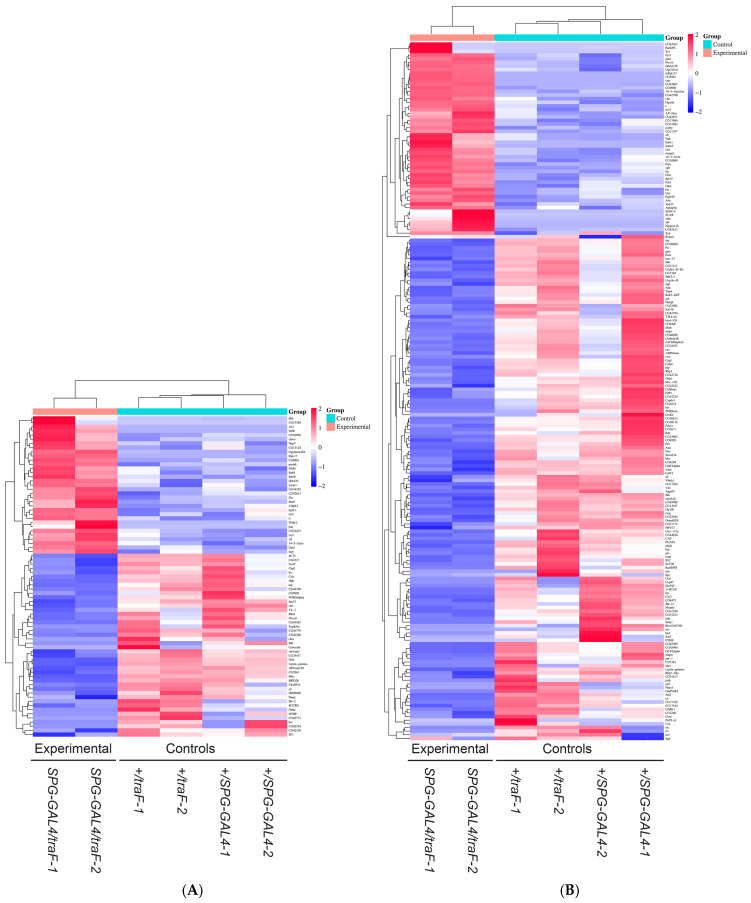
Subset of RNAs previously identified as tra regulated (**A**) compared to the rest of identified differentially expressed RNAs (**B**). Heatmaps showing transcripts with changes in gene expression in isolated brains of control males (*w^1118^*; *+*; *SPG-GAL4/+* and *w^1118^*; *+ UAS-traF/+*, respectively) and males with feminized BBB (*w^1118^*; *+*; *SPG-GAL4/UAS-traF*). Red to blue shadings represent higher and lower relative expression levels, respectively.

To examine the sex-specific role of the BBB in the regulation of brain gene expression we used the *GAL4/UAS* system to specifically feminize BBB cells in otherwise normal males. We used a BBB-specific GAL4 driver (*SPG-GAL4*) [16,32] to express *UAS-traF*. We have previously shown that these males have significantly reduced courtship [31]. The brains of adult mature males were isolated by dissection. Control males containing only *SPG-GAL4* or only *UAS-traF* were grown, aged and dissected in parallel to control for potential effects of these transgenes alone. We subsequently examined whole brain poly-A+ transcripts by RNA sequencing (File S1) and identified 283 transcripts that were differentially expressed (Figure 1, File S2). Among the differentially expressed transcripts identified in the *w^1118^*; *+*; *SPG-GAL4/UAS-traF* experimental flies were 77 transcripts that had previously been found to be *tra* dependent by Chang et al. [36], validating our approach and indicating that the differences we observe are in response to experimental TraF expression (Figure 2A). The other transcripts that were not previously described as *tra* dependent show a similar expression profile as shown by heatmap (Figure 2B). These results demonstrate a change in RNA expression in response to feminization of the BBB and identifie RNAs that might mediate the sex-specific BBB/courtship circuit interactions for the regulation of male courtship behavior. 

**Figure 3:**
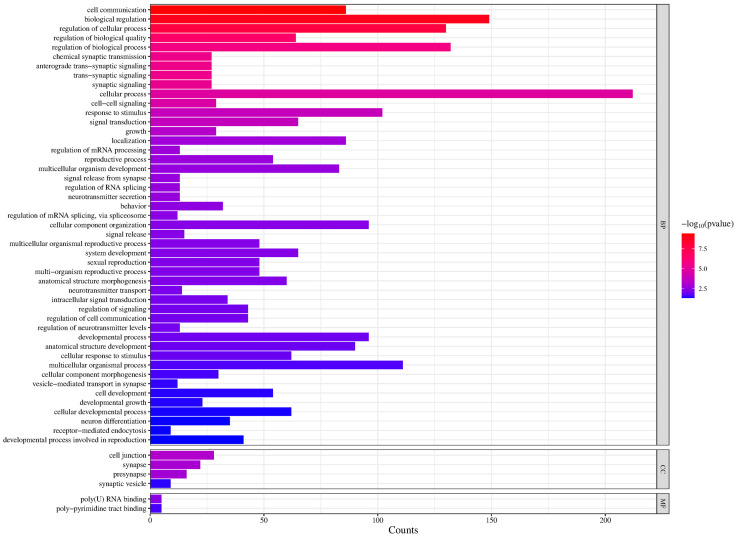
*Enriched GO categories of differentially expressed RNAs*. Enriched categories as determined by FlyMine, with number of differentially expressed genes in these categories indicated.

GO analysis of the differentially expressed RNAs revealed enrichment in signaling, cell communication, signal transduction, synaptic communication and cell junction properties (Figure 3). Identified RNAs include transcripts consistent with BBB function, such as cytoskeletal components (for example *moe* and *shot*), as well as its known physiological function (for example the *Glut1* glucose transporter). Several RNAs that are implied in Juvenile Hormone (JH) and Ecdysone hormonal signaling, as well as for insulin signaling, were identified as well, suggesting that hormonal processes are responsive to the sex-specific characteristics of BBB cells. A number of transcripts are implied in synaptic and neural function, indicating an effect on neuronal function.

**Figure 4:**
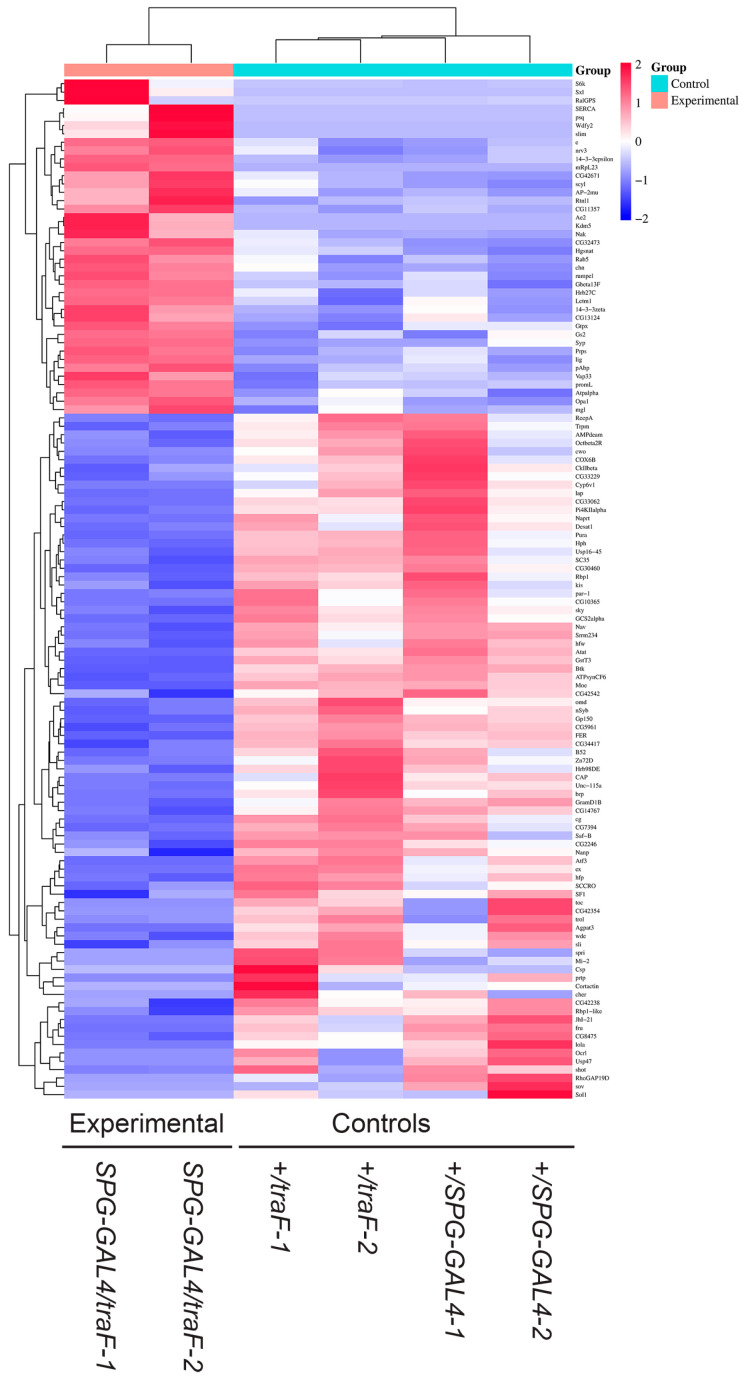
*Subset of BBB transcripts that change upon feminization of SPG cells*. Heatmap showing transcripts with changes in gene expression in isolated brains of control males (*w^1118^*; *+*; *SPG-GAL4/+* and *w^1118^*; *+ UAS-traF/+*, respectively) and males with feminized BBB (*w^1118^*; *+*; *SPG-GAL4/UAS-traF*). Red to blue shadings represent higher and lower relative expression levels, respectively.

**Figure 5:**
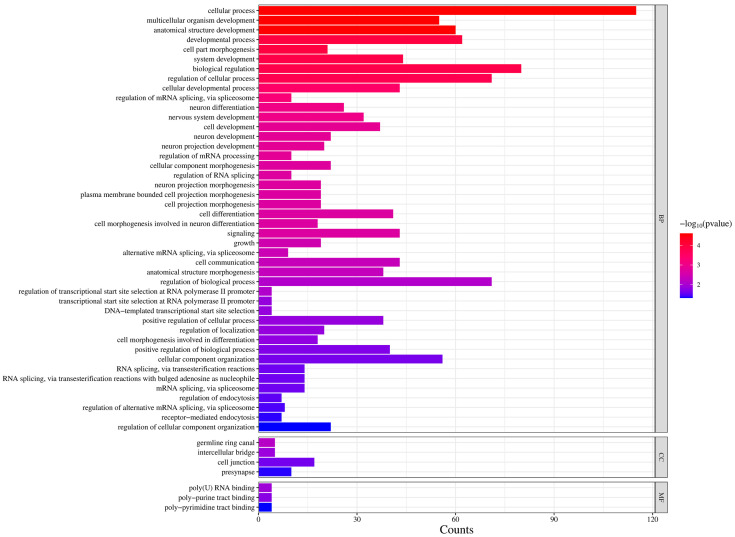
*Enriched GO categories of TraF-regulated RNAs found in common with a SPG cell transcript library*. Enriched categories as determined by FlyMine, with number of differentially expressed genes in these categories indicated.

In our experiments, we identified RNAs in whole dissected brains following BBB feminization and identified 283 differentially expressed transcripts. To assess how many of them might be BBB transcripts, and whether non-BBB transcripts might be affected by BBB feminization, we compared our set of differentially expressed RNAs to a previously described BBB transcriptome. Contreras et al. (2021) [25] used targeted DamID to examine the effect of nutritional status on BBB and neuroblast gene expression. They profiled SPG cells of the BBB under different conditions. We combined their SPG datasets to obtain one set of SPG-specific transcripts and compared it to our list of TraF-regulated RNAs. We identified overlaps for 125 transcripts, identifying them as BBB transcripts that change upon feminization of SPG cells (Figure 4, File S3; the transcripts are also marked in Figure 1 and File S2). GO analysis (Figure 5) of this subset shows signatures of cell development and differentiation as well as cellular processes regulation. RNA splicing and processing is another significant category. TraF is a splicing regulator and is part of a sex-specific splicing cascade. Differentially spliced RNAs in the BBB may thus be mediators of sex-specific brain interaction. As expected, transcripts identified as present in SPG cells did not show signatures of synaptic activity and neuronal connections that we see in the entire dataset of TraF regulated genes. A total of 158 TraF regulated transcripts did not overlap with the SPG reference library, suggesting that they represent brain transcripts that change through indirect effects in response to BBB feminization. 

## 4. Discussion

Our experiments examined changes that occur in the brain transcriptome following genetic feminization of BBB cells in otherwise normal males. These are the same genotypes and conditions that resulted in significantly reduced male courtship behavior [31]. We selectively feminized BBB cells by expression of TraF and examined the effect on RNAs that are present in whole dissected brains. We analyzed dissected brains containing an intact BBB. The observed changes therefore reflect effects of TraF expression in BBB cells, but likely also identify the effect BBB feminization has on the brain transcriptome. In agreement with this, GO analysis shows enrichment in transcripts involved in cell junction properties and synaptic communication. A subsequent comparison with a SPG transcriptome further supports this hypothesis. In agreement with effects in the BBB, we see changes in *Glut1*, a BBB Glucose transporter, as well as transcripts implicated in transmembrane transport. We also find RNAs classified as “cell junction” related. Regulated tight junctions and cytoskeleton are hallmarks of BBB cells. For example, Moe (encoded by *CG10710*) is an actin binding protein, and Shot (encoded by *CG18076*) has been implicated in microtubule/cytoskeleton organization and actin binding. This is intriguing since the establishment of BBB integrity during development relies on correct actin cytoskeleton organization [23,37]. While tight junctions and barrier are firmly established and essential in adults, there is evidence that selective small changes in permeability (that do not compromise general barrier function) may underlie some BBB functions such as sleep with subtle changes in barrier permeability [28,29,37]. While we would expect transcriptional changes in the BBB following expression of TraF in these cells, transcripts in neurons and non-BBB glia may also change in response to feminization of the BBB. BBB processes that affect behavior require communication with the brain circuits that regulate the behavioral output. Indeed, GO analysis of Biological Processes (Figure 3) shows enrichment in signaling, cell communication, signal transduction and synaptic communication. Comparison to a SPG RNA dataset showed 158 transcripts that did not overlap with SPG transcripts present in this database. The 158 transcripts likely reflect indirect effects following BBB feminization that might be caused by changes in BBB permeability or in response to altered physiological processes in BBB cells. They are potential candidates for BBB/brain interaction. We have previously shown that *Dop2R*, a dopamine receptor, is expressed and required in the BBB for courtship [38]. Other receptors identified in our screen (for example the *5-HT1B* serotonin receptor and the *Octopamineβ2R*) are candidates to be tested for similar roles. 

Several differentially expressed RNAs are involved in hormone signaling. We have previously shown that Juvenile Hormone, a hormone best known for its developmental role, is required in mature males for courtship [39]. When JH synthesis was disrupted in mature males by knockdown of JHAMT, an essential enzyme in its biosynthesis, courtship was significantly reduced. The defect could be rescued by application of Methoprene, a JH analog. It is intriguing that *Juvenile Hormone Inducible 21* (*JHI-21*) was identified in our screen. It is predicted to enable transmembrane transport as a large neutral amino acid transporter. Li et al. have suggested that in adults it is required in the BBB for the regulation of sleep in *Drosophila* [30]. Furthermore, our screen identified *ETHR* (*Ecdysis triggering hormone receptor*), a GPCR involved in JH and Ecdysone signaling and the receptor for ETH. In development, ETH (Ecdysis triggering Hormone) is induced by Ecdysone and regulates ecdysis. In adults, it has been shown to be needed in adult males for courtship inhibition after completion of copulation. The mutant led to a reversal of post-copulation courtship inhibition (PCCI) and increased male-male courtship. The same phenotypes were observed for ETHR mutants. ETHR silencing in the *corpora allata*, the source of Juvenile Hormone, also relieves post-copulatory inhibition [40]. Our screen has further identified *chico*, an insulin receptor substrate. *S6 kinase* and the MAP kinase *rolled* were also found, both part of the mTor signaling pathway that, among many other processes, mediates Insulin signaling These results suggest involvement of Insulin signaling and potential crosstalk with JH in the regulation of male courtship behavior that is modulated by processes in the BBB. The RNA for another brain hormone, *Diuretic hormone 44* (Dh44), showed changed levels as well. Significantly, the screen has also identified several RNAs that are known to be developmentally regulated by Ecdysone, the other major developmental insect hormone. The transcripts for *Ecdysone-induced protein 78C* (*Eip78C*) and *Ecdysone-induced protein 93F* (*Eip93F*), both orphan nuclear receptors, as well as for *halfway* (*hfw*) with a role in the response to Ecdysone, all significantly changed in response to BBB feminization. These findings suggest a sex-specific role for Ecdysone in adult males that is regulated by the BBB. Data from Hindle et al. [41] suggest that Ecdysone and the Ecdysone receptor (EcR) are present in the BBB. We have likewise found in a microarray analysis of BBB cells of males and females that *EcR* RNA is present in SPG cells in a non-sex-specific manner [32]. Dalton et al. have shown fairly widespread expression of EcR, including its presence in *fruitless* neurons. When they reduced EcR in these neurons, they observed male-male courtship but no changes in male-female courtship [42]. Whether our current findings reflect an effect of TraF expression on Ecdysone signaling in the BBB itself or in other brain cells remains to be seen. It will be of interest to examine whether the knockdown of the TraF dependent Ecdysone mediators identified here will affect male-female or/and male-male courtship in response to knockdown in either BBB, other glial cells, or in neurons.

A total of 64 identified RNAs have no known functions or roles (22%). It is intriguing to speculate that they represent genes that act in small subsets of cells that have not been widely studied yet molecularly, such as the BBB. This finding is interesting since a possible limitation of our approach is its sensitivity in cases where gene expression changes in a subset of cells, while expression in other cell populations (that may be more abundant) may not be affected. 

In summary, we have found that changing the sexual identity of BBB cells of males affects the expression of numerous transcripts in isolated brains. Our results add to a growing list of findings demonstrating the many important roles for the BBB. In future experiments, validation of these data by an independent method such as qPCR will help identify candidates for follow-up. The effect of knocking down candidate genes in the BBB, in neurons or in other brain glia on courtship will help us understand potential roles of these genes in the regulation of courtship. These experiments will also help us understand which cells mediate these functions. The results presented here, and the future functional analysis of identified candidates will likely enhance our understanding of the mechanisms that underlie BBB / brain interactions in the control of complex behaviors.

## Data Availability

The data discussed in this publication have been deposited in NCBI’s Gene Expression Omnibus and are accessible through GEO Series accession number GSE302328. All other contributions presented in this study are included in the article/Supplementary Material. Further inquiries can be directed to the corresponding author(s).

## Supplementary Materials

The following supporting information can be downloaded at: https://www.mdpi.com/article/10.3390/cimb47080626/s1.
